# 1,1,3-Trimethyl-3-phenyl­indane

**DOI:** 10.1107/S1600536808009690

**Published:** 2008-04-16

**Authors:** Jian Men, Mei-Jia Yang, Yan Jiang, Hua Chen, Guo-Wei Gao

**Affiliations:** a College of Chemistry, Sichuan University, Chengdu 610064, People’s Republic of China

## Abstract

In the title compound, C_18_H_20_, the five-membered ring of the indane fragment adopts an envelope conformation, with the flap atom deviating by 0.399 (3) Å from the plane of the remaining four atoms. The dihedral angle between the phenyl ring and the indane benzene ring is 79.58 (7)°.

## Related literature

For related literature, see: Bateman & Gordon (1974 ▶, 1976 ▶); Ghosh & Mittal (1996 ▶); Feger *et al.* (1989 ▶).
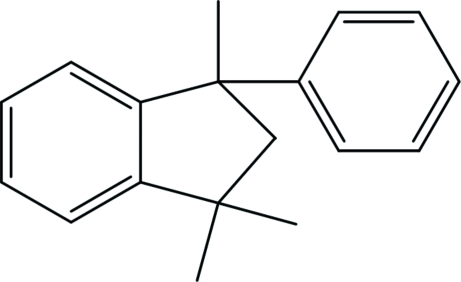


## Experimental

### 

#### Crystal data

C_18_H_20_
*M* *_r_* = 236.34
Triclinic, 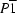

*a* = 8.192 (2) Å
*b* = 8.426 (3) Å
*c* = 11.113 (4) Å
α = 69.30 (3)°
β = 79.44 (5)°
γ = 80.37 (2)°
*V* = 701.0 (7) Å^3^
*Z* = 2
Mo *K*α radiation
μ = 0.06 mm^−1^
*T* = 291 (2) K
0.46 × 0.44 × 0.42 mm

#### Data collection

Enraf–Nonius CAD-4 diffractometer
Absorption correction: none
3682 measured reflections
2582 independent reflections
1770 reflections with *I* > 2σ(*I*)
*R*_int_ = 0.007
3 standard reflections every 200 reflections intensity decay: 0.7%

#### Refinement

*R*[*F* ^2^ > 2σ(*F* ^2^)] = 0.040
*wR*(*F* ^2^) = 0.117
*S* = 1.06
2582 reflections
170 parameters
H-atom parameters constrained
Δρ_max_ = 0.17 e Å^−3^
Δρ_min_ = −0.14 e Å^−3^

### 

Data collection: *DIFRAC* (Gabe & White, 1993 ▶); cell refinement: *DIFRAC*; data reduction: *NRCVAX* (Gabe *et al.*, 1989 ▶); program(s) used to solve structure: *SHELXS97* (Sheldrick, 2008 ▶); program(s) used to refine structure: *SHELXL97* (Sheldrick, 2008 ▶); molecular graphics: *ORTEP-3 for Windows* (Farrugia, 1997 ▶); software used to prepare material for publication: *SHELXL97*.

## Supplementary Material

Crystal structure: contains datablocks global, I. DOI: 10.1107/S1600536808009690/gk2139sup1.cif

Structure factors: contains datablocks I. DOI: 10.1107/S1600536808009690/gk2139Isup2.hkl

Additional supplementary materials:  crystallographic information; 3D view; checkCIF report

## Figures and Tables

**Fig. 1.**
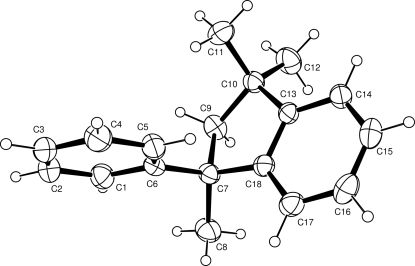
The molecular structure of the title compound with displacement ellipsoids drawn at the 30% probability level.

## supplementary crystallographic information

### Comment

Polyimides are well known for possessing excellent thermal and oxidative stability, as well as excellent mechanical properties (Ghosh & Mittal, 1996; Feger *et al.*,  1989). Furthermore, polyimides with phenylindane diamines and/or dianhydrides incorporated into the polyimide backbone have been found to be soluble in high concentration in polar organic solvents (Bateman & Gordon, 1974). Phenylindane diamines are prepared by a process comprising acid-catalyzed dimerization of α-methylstyrene and subsequent nitration and reduction of the 1,1,3-trimethyl-3-phenyl-2,3-dihydro-1*H*-indene (Bateman & Gordon, 1976).

The molecule of the title compound is shown in Fig. 1. Rings A (C1–C6) and B (C13–C18) are planar and form dihedral angle of 79.58 (7)°. The B ring forms dihedral angle of 25.38 (14)° with the plane defined by the indane Csp3 atoms C7, C9 and C10.

### Experimental

α-Methylstyrene (32.0 g, 0.30 mol) was added to a 500 ml flask equipped with a condenser and a mechanical stirrer, followed by slow addition of a previously prepared mixture of H_2_SO_4_ (68 ml) and H_2_O (130 ml). The reaction mixture was refluxed for 20 h. After it was cooled to room temperature, the lower acid phase was drawn off and discarded. The organic phase containing the phenylindane was washed with water several times. The product was recrystallized from methanol that afforded white crystals (24 g, yield 68%, m.p. 323–324 K).

### Refinement

H atoms were positioned geometrically (C—H = 0.93–0.97 Å) and refined using a riding model, with *U*_iso_(H) = 1.2*U*_eq_ (aromatic, methylene) or *U*_iso_(H) = 1.5*U*_eq_(methyl).

### Crystal data

**Table:**

C_18_H_20_	*Z* = 2
*M**_r_* = 236.34	*F*_000_ = 256
Triclinic, *P*1	*D*_x_ = 1.120 Mg m^−^^3^
Hall symbol: -P 1	Mo *K*α radiation λ = 0.71073 Å
*a* = 8.192 (2) Å	Cell parameters from 28 reflections
*b* = 8.426 (3) Å	θ = 4.4–7.7º
*c* = 11.113 (4) Å	µ = 0.06 mm^−^^1^
α = 69.30 (3)º	*T* = 291 (2) K
β = 79.44 (5)º	Block, colourless
γ = 80.37 (2)º	0.46 × 0.44 × 0.42 mm
*V* = 701.0 (7) Å^3^	

### Data collection

**Table:**

Enraf–Nonius CAD-4 diffractometer	*R*_int_ = 0.007
Radiation source: fine-focus sealed tube	θ_max_ = 25.5º
Monochromator: graphite	θ_min_ = 2.6º
*T* = 291(2) K	*h* = −9→9
ω/2τ scans	*k* = −3→10
Absorption correction: none	*l* = −12→13
3682 measured reflections	3 standard reflections
2582 independent reflections	every 200 reflections
1770 reflections with *I* > 2σ(*I*)	intensity decay: 0.7%

### Refinement

**Table:**

Refinement on *F*^2^	Hydrogen site location: inferred from neighbouring sites
Least-squares matrix: full	H-atom parameters constrained
*R*[*F*^2^ > 2σ(*F*^2^)] = 0.040	*w* = 1/[σ^2^(*F*_o_^2^) + (0.0549*P*)^2^ + 0.0915*P*] where *P* = (*F*_o_^2^ + 2*F*_c_^2^)/3
*wR*(*F*^2^) = 0.117	(Δ/σ)_max_ < 0.001
*S* = 1.06	Δρ_max_ = 0.17 e Å^−^^3^
2582 reflections	Δρ_min_ = −0.14 e Å^−^^3^
170 parameters	Extinction correction: SHELXL97 (Sheldrick, 2008), Fc^*^=kFc[1+0.001xFc^2^λ^3^/sin(2θ)]^-1/4^
Primary atom site location: structure-invariant direct methods	Extinction coefficient: 0.154 (10)
Secondary atom site location: difference Fourier map	

### Special details

**Table:**

Experimental. ^1^H NMR (400 MHz, CDCl_3_): δ = 1.03, 1.35, 1.69 (s, 3H, –CH_3_), 2.21 and 2.40 (d, 2H, –CH_2_–), 7.11–7.29 (m, 9H, Ar—H).
Geometry. All e.s.d.'s (except the e.s.d. in the dihedral angle between two l.s. planes) are estimated using the full covariance matrix. The cell e.s.d.'s are taken into account individually in the estimation of e.s.d.'s in distances, angles and torsion angles; correlations between e.s.d.'s in cell parameters are only used when they are defined by crystal symmetry. An approximate (isotropic) treatment of cell e.s.d.'s is used for estimating e.s.d.'s involving l.s. planes.
Refinement. Refinement of *F*^2^ against ALL reflections. The weighted *R*-factor *wR* and goodness of fit *S* are based on *F*^2^, conventional *R*-factors *R* are based on *F*, with *F* set to zero for negative *F*^2^. The threshold expression of *F*^2^ > 2σ(*F*^2^) is used only for calculating *R*-factors(gt) *etc*. and is not relevant to the choice of reflections for refinement. *R*-factors based on *F*^2^ are statistically about twice as large as those based on *F*, and *R*-factors based on ALL data will be even larger.

### Fractional atomic coordinates and isotropic or equivalent isotropic displacement parameters (Å^2^)

**Table:**

	*x*	*y*	*z*	*U*_iso_*/*U*_eq_	
C18	0.39195 (18)	0.00422 (18)	0.21135 (14)	0.0428 (4)	
C6	0.20481 (17)	0.27044 (18)	0.22736 (14)	0.0436 (4)	
C14	0.6116 (2)	−0.1842 (2)	0.32179 (15)	0.0527 (4)	
H14	0.7136	−0.2011	0.3526	0.063*	
C13	0.54458 (18)	−0.02135 (18)	0.25548 (14)	0.0421 (4)	
C7	0.34020 (18)	0.19224 (19)	0.14280 (14)	0.0453 (4)	
C17	0.3069 (2)	−0.1345 (2)	0.23220 (17)	0.0567 (4)	
H17	0.2043	−0.1181	0.2025	0.068*	
C10	0.61857 (19)	0.14541 (19)	0.22095 (15)	0.0478 (4)	
C1	0.1330 (2)	0.4375 (2)	0.17672 (18)	0.0595 (5)	
H1	0.1670	0.5006	0.0908	0.071*	
C5	0.1502 (2)	0.1821 (2)	0.35573 (16)	0.0542 (4)	
H5	0.1951	0.0697	0.3928	0.065*	
C3	−0.0391 (2)	0.4221 (3)	0.3779 (2)	0.0665 (5)	
H3	−0.1201	0.4724	0.4279	0.080*	
C15	0.5266 (2)	−0.3214 (2)	0.34208 (17)	0.0606 (5)	
H15	0.5716	−0.4313	0.3863	0.073*	
C2	0.0126 (2)	0.5121 (2)	0.2504 (2)	0.0696 (5)	
H2	−0.0339	0.6240	0.2137	0.083*	
C4	0.0303 (2)	0.2575 (3)	0.43009 (18)	0.0653 (5)	
H4	−0.0034	0.1957	0.5164	0.078*	
C9	0.5091 (2)	0.2673 (2)	0.11982 (16)	0.0543 (4)	
H9A	0.5650	0.2782	0.0329	0.065*	
H9B	0.4892	0.3795	0.1289	0.065*	
C16	0.3754 (2)	−0.2966 (2)	0.29712 (18)	0.0640 (5)	
H16	0.3192	−0.3899	0.3107	0.077*	
C8	0.2796 (2)	0.2204 (2)	0.01297 (16)	0.0664 (5)	
H8A	0.3617	0.1654	−0.0374	0.100*	
H8B	0.2635	0.3405	−0.0340	0.100*	
H8C	0.1759	0.1728	0.0293	0.100*	
C11	0.5994 (3)	0.1997 (2)	0.34135 (19)	0.0690 (5)	
H11A	0.4832	0.2128	0.3749	0.104*	
H11B	0.6446	0.3062	0.3180	0.104*	
H11C	0.6585	0.1139	0.4065	0.104*	
C12	0.8028 (2)	0.1333 (3)	0.1630 (2)	0.0723 (6)	
H12A	0.8426	0.2429	0.1377	0.109*	
H12B	0.8146	0.0980	0.0883	0.109*	
H12C	0.8669	0.0513	0.2266	0.109*	

### Atomic displacement parameters (Å^2^)

**Table:**

	*U*^11^	*U*^22^	*U*^33^	*U*^12^	*U*^13^	*U*^23^
C18	0.0409 (8)	0.0477 (9)	0.0410 (8)	−0.0052 (7)	−0.0006 (6)	−0.0185 (7)
C6	0.0386 (8)	0.0477 (9)	0.0461 (9)	−0.0021 (7)	−0.0125 (7)	−0.0155 (7)
C14	0.0555 (10)	0.0501 (10)	0.0506 (9)	0.0029 (8)	−0.0100 (8)	−0.0171 (8)
C13	0.0434 (8)	0.0441 (8)	0.0388 (8)	−0.0017 (6)	−0.0031 (6)	−0.0162 (7)
C7	0.0423 (8)	0.0506 (9)	0.0411 (8)	−0.0023 (7)	−0.0078 (6)	−0.0131 (7)
C17	0.0481 (9)	0.0637 (11)	0.0656 (11)	−0.0115 (8)	−0.0018 (8)	−0.0309 (9)
C10	0.0422 (8)	0.0472 (9)	0.0537 (9)	−0.0060 (7)	−0.0078 (7)	−0.0155 (7)
C1	0.0623 (11)	0.0537 (10)	0.0595 (11)	−0.0005 (8)	−0.0128 (9)	−0.0157 (8)
C5	0.0530 (10)	0.0568 (10)	0.0482 (9)	0.0022 (8)	−0.0064 (8)	−0.0158 (8)
C3	0.0483 (10)	0.0852 (14)	0.0811 (14)	0.0089 (9)	−0.0138 (9)	−0.0509 (12)
C15	0.0738 (12)	0.0422 (9)	0.0581 (10)	−0.0012 (8)	−0.0002 (9)	−0.0138 (8)
C2	0.0668 (12)	0.0601 (11)	0.0882 (15)	0.0127 (9)	−0.0238 (11)	−0.0348 (11)
C4	0.0590 (11)	0.0837 (14)	0.0536 (10)	−0.0014 (10)	−0.0020 (9)	−0.0289 (10)
C9	0.0491 (9)	0.0510 (9)	0.0525 (10)	−0.0069 (7)	−0.0026 (7)	−0.0063 (8)
C16	0.0715 (12)	0.0493 (10)	0.0735 (12)	−0.0191 (9)	0.0083 (10)	−0.0267 (9)
C8	0.0659 (12)	0.0851 (13)	0.0473 (10)	0.0056 (10)	−0.0148 (9)	−0.0236 (9)
C11	0.0802 (13)	0.0639 (11)	0.0758 (13)	−0.0119 (9)	−0.0212 (10)	−0.0317 (10)
C12	0.0464 (10)	0.0720 (12)	0.0943 (15)	−0.0103 (9)	−0.0070 (10)	−0.0219 (11)

### Geometric parameters (Å, °)

**Table:**

C18—C13	1.382 (2)	C5—H5	0.9300
C18—C17	1.388 (2)	C3—C4	1.368 (3)
C18—C7	1.520 (2)	C3—C2	1.375 (3)
C6—C5	1.384 (3)	C3—H3	0.9300
C6—C1	1.388 (2)	C15—C16	1.376 (3)
C6—C7	1.536 (2)	C15—H15	0.9300
C14—C15	1.379 (2)	C2—H2	0.9300
C14—C13	1.382 (2)	C4—H4	0.9300
C14—H14	0.9300	C9—H9A	0.9700
C13—C10	1.519 (2)	C9—H9B	0.9700
C7—C8	1.538 (2)	C16—H16	0.9300
C7—C9	1.558 (2)	C8—H8A	0.9600
C17—C16	1.377 (3)	C8—H8B	0.9600
C17—H17	0.9300	C8—H8C	0.9600
C10—C12	1.530 (2)	C11—H11A	0.9600
C10—C11	1.535 (3)	C11—H11B	0.9600
C10—C9	1.539 (2)	C11—H11C	0.9600
C1—C2	1.378 (3)	C12—H12A	0.9600
C1—H1	0.9300	C12—H12B	0.9600
C5—C4	1.385 (2)	C12—H12C	0.9600
			
C13—C18—C17	119.85 (15)	C16—C15—C14	120.29 (16)
C13—C18—C7	111.75 (13)	C16—C15—H15	119.9
C17—C18—C7	128.40 (14)	C14—C15—H15	119.9
C5—C6—C1	116.91 (16)	C3—C2—C1	120.35 (18)
C5—C6—C7	122.83 (14)	C3—C2—H2	119.8
C1—C6—C7	120.26 (15)	C1—C2—H2	119.8
C15—C14—C13	119.63 (16)	C3—C4—C5	120.67 (18)
C15—C14—H14	120.2	C3—C4—H4	119.7
C13—C14—H14	120.2	C5—C4—H4	119.7
C14—C13—C18	120.23 (15)	C10—C9—C7	108.20 (13)
C14—C13—C10	128.01 (14)	C10—C9—H9A	110.1
C18—C13—C10	111.76 (14)	C7—C9—H9A	110.1
C18—C7—C6	112.36 (13)	C10—C9—H9B	110.1
C18—C7—C8	111.23 (14)	C7—C9—H9B	110.1
C6—C7—C8	109.72 (13)	H9A—C9—H9B	108.4
C18—C7—C9	100.82 (13)	C15—C16—C17	120.42 (16)
C6—C7—C9	111.63 (13)	C15—C16—H16	119.8
C8—C7—C9	110.83 (14)	C17—C16—H16	119.8
C16—C17—C18	119.56 (16)	C7—C8—H8A	109.5
C16—C17—H17	120.2	C7—C8—H8B	109.5
C18—C17—H17	120.2	H8A—C8—H8B	109.5
C13—C10—C12	112.22 (14)	C7—C8—H8C	109.5
C13—C10—C11	110.21 (14)	H8A—C8—H8C	109.5
C12—C10—C11	109.67 (15)	H8B—C8—H8C	109.5
C13—C10—C9	101.46 (13)	C10—C11—H11A	109.5
C12—C10—C9	111.39 (15)	C10—C11—H11B	109.5
C11—C10—C9	111.70 (15)	H11A—C11—H11B	109.5
C2—C1—C6	121.72 (18)	C10—C11—H11C	109.5
C2—C1—H1	119.1	H11A—C11—H11C	109.5
C6—C1—H1	119.1	H11B—C11—H11C	109.5
C6—C5—C4	121.37 (17)	C10—C12—H12A	109.5
C6—C5—H5	119.3	C10—C12—H12B	109.5
C4—C5—H5	119.3	H12A—C12—H12B	109.5
C4—C3—C2	118.96 (18)	C10—C12—H12C	109.5
C4—C3—H3	120.5	H12A—C12—H12C	109.5
C2—C3—H3	120.5	H12B—C12—H12C	109.5
